# Evaluation of the toxicity and hypoglycemic effect of the aqueous extracts of *Cnidoscolus quercifolius* Pohl

**DOI:** 10.1590/1414-431X20176361

**Published:** 2017-08-31

**Authors:** S.M. Lira, N.V. Canabrava, S.R. Benjamin, J.Y.G. Silva, D.A. Viana, C.L.S. Lima, P.F.M. Paredes, M.M.M. Marques, E.O. Pereira, E.A.M. Queiroz, M.I.F. Guedes

**Affiliations:** 1 Laboratório de Biotecnologia e Biologia Molecular, Universidade Estadual do Ceará, Fortaleza, CE, Brasil; 2 Laboratório de Patologia Clínica Pathovet, Fortaleza, CE, Brasil; 3 Laboratório de Parasitologia e Ecologia de Doenças Negligenciadas, Universidade Federal do Piauí, Picos, PI, Brasil

**Keywords:** Faveleira, Diabetes mellitus, Medicinal plants, Hypoglycemic activity, Aqueous extracts

## Abstract

Diabetes mellitus is one of the most common chronic degenerative diseases, and it is estimated to increase worldwide to around 415 million and to impact 642 million in 2040. Research shows that some plants are sources of bioactive compounds against diabetes. Thus, the objective of this work was to evaluate the oral toxicity and the hypoglycemic effect of the aqueous extract of the leaves of *Cnidoscolus quercifolius* Pohl. Diabetes was induced in Swiss mice with streptozotocin and the mice were treated with an aqueous extract of *C. quercifolius* leaves for a period of 30 days. Phytochemical analysis showed that the extract was rich in flavonoids, catechins and triterpenoid, which did not show any mortality and behavioral alterations in mice treated with 200, 1000, and 2000 mg/kg body weight of the extract for 14 days. Histopathological analysis of organs (kidney, pancreas, liver) from mice treated with the 2000 mg/kg extract revealed no architectural change. In the present study, we found a 29% reduction in glucose levels in animals receiving 200 mg/kg body weight. These results are very promising because they showed that *C. quercifolius* had a hypoglycemic effect and did not present oral toxicity, thus being a new source of compounds for the control of diabetes.

## Introduction

Diabetes mellitus (DM) is a heterogeneous group of metabolic disorders that have in common hyperglycemia as a consequence of failure of insulin action (1). During the early stages of the disease, asymptomatic patients, especially those with type 2 diabetes mellitus (T2DM), can present stupor, coma and death due to ketoacidosis. The severity of the symptoms is related to the type and duration of diabetes (2).

It is estimated that the world population with diabetes is around 415 million and will reach 642 million in 2040 (3). In Brazil, in the period of 1998-2008, the standardized prevalence of DM increased from 2.9 to 4.3%. In 2015, there were 9.1 million people aged between 20 and 79 years with DM, which corresponds to approximately 6.2% of the adult population (4,5). Projections demonstrate that T2DM will be responsible for a remarkable share of the global disease by 2030 (6). The etiology of diabetes is multifactorial, being a product of the interplay between genetic and environmental factors, as well as dietary and lifestyle factors (1).

The use of herbal medicines, medicinal plants and phytonutrients continues to expand rapidly across the world, with an estimated 80% of the world's population using this type of medication, especially in developing countries (7,8).

The World Health Organization established guidelines for the evaluation of herbal medicines, defining some criteria for assessing the quality, safety and efficacy of plants (9).

Brazil has huge potential for the disclosure of new bioactive substances, standing out amongst countries with the most diverse flora, despite the fact that efficacy of some substances has not been tested pharmacologically (10). The present demand for complementary and alternative medicine may be in part due to the inadequate knowledge of traditional medicine or the high cost and side effects of manufactured drugs. In the last decades, the use of herbal medicine has been widely embraced and accepted by the public. Herbal medicinal products have been included in healthcare and traditional medical practice in developed countries, mainly in UK and Europe (8,11,12).

Several studies have demonstrated the effect of medicinal plants that have shown to be promising in monitoring glycemia, including *Cnidoscolus chayamansa, Allium cepa, Psidium guajava, Panax ginseng, Phaseolus vulgaris, Passiflora Glandulosa,* and *Copernicia* cerifera (13–19). Among these plants, *Cnidoscolus quercifolius* Pohl. (Mart Pax et K. Hoffm.) belongs to the Euphorbiaceae family, popularly known as faveleira. It presents several biological activities such as antitumor effects, through the neofavelone compound obtained from the plant bark (20). The ethanolic extract of bark and leaves show anti-inflammatory activity and antinociceptive activity (21); antimicrobial, antifungal effects were found with acetyl cholinesterase, an antioxidant from the leaves, roots, and barks (22). However, there are no studies demonstrating the hypoglycemic effect of *C. quercifolius*. Thus, the objective of this work was to evaluate the toxicity and the hypoglycemic effect of the aqueous extracts of *C. quercifolius* leaves in the treatment of streptozotocin-induced diabetic mice.

## Material and Methods

### Plant material and preparation of extracts

*C. quercifolius* Pohl. (Mart. Pax et K. Hoffm.) was collected from its natural habitat in the city of Fortaleza, CE (Northeastern Brazil), and identified by a botanist of the Prisco Bezerra Herbarium (Universidade Federal do Ceará, UFC), where a specimen voucher was deposited (No. 56043) (22). The dried leaves (100 g) at room temperature, were boiled in water for 5 min. The solution was then filtered through celite and lyophilized. The extract was weighed and stored in a container at 6°C until use.

### Phytochemical analysis

The extracts were subjected to phytochemical screening, following the protocols described by Matos (23). Chemical tests were performed using specific reagents, observing color changes or formation of a precipitate, and characteristic for each class of substances. Tests were performed for the detection of phenols, flavones, flavonols, xanthones, catechins, anthocyanins, anthocyanidins, triterpenoids, flavanones, saponins, alkaloids, and tannins.

### Animals

Male and female Swiss mice (*Mus musculus*) aged between 8 and 12 weeks (25.0-30.0 g), were obtained from the vivarium of the UFC. The animals were kept in polypropylene cages at room temperature between 24° and 25°C in light-dark cycles of 12/12 h. All animals received water and food ad *libitum*. The Ethical Committee on Animal Research of the Universidade Estadual do Ceará approved the experimental protocol (No. 1606145-2015).

### Acute toxicity

For the acute toxicity study, 28 Swiss male mice and 28 female mice weighing between 25-30 g, were used. Animals were divided into 2 groups (n=7): normal control treated orally with water (NC), and mice treated orally with aqueous extract of faveleira leaves (AEF; 200, 1000, 2000 mg/kg). All animals were fasted for 4 h, and then received food and water again after the administration of the extracts. Following treatment, the mice were observed at 30, 60, 120, 240, and 360 min and every 24-h during a 14-day period. During examination, the following parameters were assessed: heart rate, respiratory rate, number of deaths and side effects (e.g., piloerection, diarrhea, sialorrhea, hypnosis and seizures) (24). After behavioral observation and at the end of this period all animals were euthanized by cervical dislocation and the kidney, pancreas, and liver were harvested and weighed.

The kidney, pancreas, and liver were used for the histopathological analysis. The isolated fragments were fixed in 10% neutral formalin and placed in paraffin blocks for conventional histological processing (25). Then, 5-µm sections were obtained and stained with hematoxylin-eosin (HE). The slides were examined for the identification of histological alterations with conventional optical microscopy (Nikon YS2, Nikon, Japan), and images representative of each organ were captured with a digital camera (Nikon Coolpix L14 7.1 megapixels, Nikon).

### Diabetes induction

Diabetes mellitus was induced by a single intraperitoneal (*ip*) injection of streptozotocin (STZ; Sigma¯, USA) at the high dose of 140 mg/kg in 12-h fasting mice (26). Animals with blood glucose levels equal to or greater than 200 mg/dL were considered to be diabetic.

### Experimental protocol

The animals were divided into the following 4 groups (n=6). Normal control group (NC): healthy mice treated with water (0.2 mL water·day^-1^·animal^-1^); MET 200 group: diabetic mice treated with metformin at 200 mg/kg body weight diluted in water; AEF 100 group: diabetic mice treated with aqueous extract of faveleira leaves at 100 mg/kg body weight diluted in water; AEF 200 group: diabetic mice treated with aqueous extract of faveleira leaves at 200 mg/kg body weight diluted in water). Animals received doses orally for a period of 30 consecutive days, as previously reported by Barbosa et al. (27), with some modifications.

### Determination blood glucose

Blood was collected through the retro-orbital plexus using a capillary tube on days 0, 10, 20, and 30 to determine the glucose concentration. The Metrolab (Romenia) 23300 version 1.7 device was used, which uses the kinetic method for serum samples.

The serum was subjected to glucose analysis by commercial kits using the manufacturer's technical recommendations (Bioclin¯, Brazil).

### Statistical analysis

Data are reported as means±SD. Statistical significance of differences between groups was assessed using one-way ANOVA, followed by the Tukey test. P<0.05 was considered to be significant.

## Results

The phytochemical analysis of *C. quercifolius* aqueous extracts revealed the presence of phenols, flavones, flavonols, xanthones, catechins, triterpenoids, and tannins.

For the acute *in vivo* toxicity test of the *C. quercifolius* aqueous extract, doses of 200, 1000, and 2000 mg/kg of animal weight were used. The results of the hippocratic screening showed that there was no motor and sensorial alterations in the animals at the doses tested, and there was no death within 14 days.

There were no significant differences (P>0.05) in liver and kidney weight between the Control group and the groups treated with aqueous extract of *C. quercifolius* at all doses, in both male and female mice (Table 1).

**Table 1.** Effect of aqueous extracts of *Cnidoscolus quercifolius* on relative weight of liver and kidneys of male and female mice.

Groups	M liver	M kidney	F liver	F kidney
NC	4.96±0.19	1.20±0.05	4.66±0.11	1.01±0.02
AEF 200	5.23±0.23	1.22±0.03	4.89±0.15	1.10±0.04
AEF 1000	5.25±0.20	1.21±0.05	4.91±0.24	1.06±0.06
AEF 2000	5.47±0.21	1.20±0.04	4.92±0.15	1.12±0.03

M liver: male liver; M kidney: male kidney; F liver: female liver; F kidney: female kidney; NC: normal control group; AEF 200: aqueous extract at the dose of 200 mg/kg; AEF 1000: aqueous extract at the dose of 1000 mg/kg; AEF 2000: aqueous extract at the dose of 2000 mg/kg. There was no significance difference between the normal control (NC) group and the groups treated with AEF (P>0.05, one way ANOVA followed by the Tukey test). Data are reported as means±SD.

**Figure 1.**
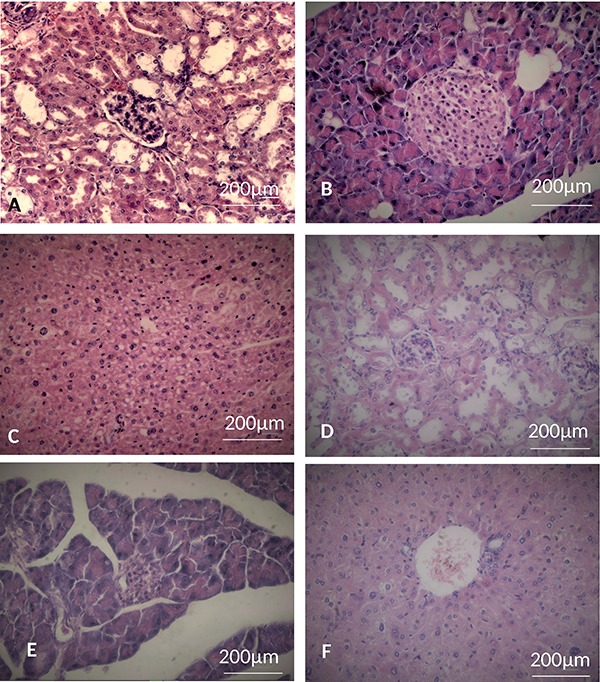
Histopathological observations of the kidney (*A*), pancreas (*B*) and liver (*C*) of mice treated with the aqueous extracts of faveleira and of the kidney (*D*), pancreas (*E*) and liver (*F*) treated with water. Hematoxylin-eosin staining.

The histopathological analysis of the organs of the animals treated with 2000 mg of the aqueous extract of *C. quercifolius* showed no architectural alteration (Figure 1A-C).

The streptozotocin-induced diabetes increased the blood glucose levels in the mice to above 200 mg/dL. The hypoglycemic action of the aqueous extract *C. quercifolius* significantly reduced blood glucose levels by 29.1% in the diabetic animals treated with 200 mg/kg body weight (P<0.05). *C. quercifolius* had a hypoglycemic effect beginning at the 20th day of treatment, and this effect increased at the end of the treatment (Table 2). There was little hypoglycemic effect at a dose of 100 mg/kg.

**Table 2.** Effect of the aqueous extracts of *Cnidoscolus quercifolius* on serologic levels of glucose in diabetic mice.

Groups	Day 0	Day 10	Day 20	Day 30	%CV
NC	105.71±8.3	107.59±9.3	100.27±12.9	106.15±5.0	+0.41
MET	296.30±24.4	246.65±54.2	280.19±43.8	206.30±24.4	-43.68
AEF 100	295.65±44.1	309.67±61.8	290.99±58.4	265.79±40.1	-10.1
AEF 200	302.22±48.2	253.31±78.5	238.47±41.4*	214.24±54.23*	-29.1

NC: normal control group; MET: metformin at the dose of 200 mg/kg; AEF 100: aqueous extract at the dose of 100 mg/kg; AEF 200: aqueous extract at the dose of 200 mg/kg. Data are reported as means±SD (n=7). There was a significant hypoglycemic effect beginning at treatment day 20 that increased at the end of treatment (*P<0.05, one-way ANOVA followed by the Tukey test).

## Discussion

The phytochemical analysis of the aqueous extract of *C. quercifolius* revealed the presence of phenols, flavones, flavonols, xanthones, catechins, triterpenoids, and tannins. Studies have shown that flavonoids, when ingested on a regular basis through diet, can help prevent chronic non-communicable diseases due to their antioxidant, anti-inflammatory, anti-hyperglycemic, anticarcinogenic and antiatherogenic effects, as well as antibacterial and antiviral activities (28,29). Triterpenoids are metabolites that also have an antidiabetic potential, which act to increase the release of insulin, modifying glucose metabolism, inhibiting hyperglycemic factors, and inhibiting or stimulating enzyme synthesis (30). Antioxidants, such as phenolic acids, flavonoids, and tannins, among others, are present in different parts of plants, and are associated with the reduction of risks of diseases such as diabetes (31).

Paredes et al. (22) conducted a study with methanolic extract of the leaf, root bark and root of *C. quercifolius* and did not observe the presence of triterpenoids. This difference in results between the aqueous and methanolic extracts of *C. quercifolius* can be justified by the solubility of the substances, which may or may not be soluble in the solvent used (32).

It is recommended that herbal medicine candidates be tested for their efficacy and safety (33). In the toxicity tests of *C. quercifolius*, there were no physical, behavioral or motor changes, or death of treated animals, as well as no changes in the weights of the organs. This is an important result, since the kidneys are responsible for numerous functions, such as reabsorption, homeostasis, filtration, endocrine and metabolic functions (34). Furthermore, liver weights did not increase, which suggests there was no hepatocellular hypertrophy (35).

According to the histopathological analysis, the aqueous extract of the faveleira did not cause architectural alterations of the liver (Figure 1C). In relation to the pancreas (Figure 1B), there was also no architectural alteration, differently from the study of Kalita et al. (36), which found that the methanolic extract of the root of *Musa balbisiana colla* regenerated the pancreatic islets of Langerhans.

These results indicate that the applied dosages were potentially safe. This is the first work that depicts the *in vivo* toxicity of *C. quercifolius,* demonstrating that its use can be safe.

This herbal drug has some advantages, such as efficacy, cost and hypoglycemic effects (37). The aqueous extract of *C. quercifolius* showed a hypoglycemic effect at a concentration of 200 mg/kg for 20 and 30 days. A previous study by Achia et al. (38), with the leaf extracts of *C. aconitifolius* in diabetic rats, showed that there was a reduction in glycemia. These results demonstrate that the genus *Cnidoscolus* has a potential hypoglycemic effect in animals.

In conclusion, the aqueous extract of *C. quercifolius* at a dose of 200 mg/kg body weight presented a hypoglycemic effect in diabetic mice and showed no toxicity. These results are very promising because they showed that *C. quercifolius* might be a new source of compounds for the treatment of diabetes.
